# The Immunopathogenesis of uveitis

**DOI:** 10.3389/fmed.2026.1717056

**Published:** 2026-03-02

**Authors:** Jimin Han, James Harper, David A. Copland, Panayiotis Maghsoudlou

**Affiliations:** 1 Academic Unit of Ophthalmology, Translational Health Sciences, Bristol, United Kingdom; 2 National Institute of Health Research (NIHR) Moorfields Biomedical Research Centre, Moorfields Eye Hospital NHS Foundation Trust and UCL Institute of Ophthalmology, London, United Kingdom

**Keywords:** autoimmunity, HLA–ERAP axis, immune privilege, inflammasome, molecular mimicry, uveitis

## Abstract

Uveitis encompasses a heterogeneous group of intraocular inflammatory disorders and remains a leading cause of preventable visual loss. Its immunopathogenesis reflects the interplay between a uniquely regulated ocular environment and triggers that breach or bypass that privilege. Much of our mechanistic understanding derives from animal models, which have helped define key features of ocular immune regulation. Layered mechanisms normally restrain inflammation: physical barriers (blood–aqueous and blood–retina), a locally immunosuppressive milieu, and systemic tolerance circuits such as anterior chamber–associated immune deviation. When these controls fail, disease emerges through a number of broad pathways. In autoimmune uveitis, genetic susceptibility (HLA class I/II and peptide-trimming enzymes such as ERAP) shapes antigen display and lowers activation thresholds for autoreactive T-cells. Antigen presentation in draining nodes primes Th1/Th17 responses. Within the eye, effector T-cells are restimulated by resident microglia and recruited macrophages, driving cytokine cascades that disrupt the blood–retina barrier and amplify leukocyte recruitment. B cells may augment tissue injury via antigen presentation, cytokine production, local antibody formation, and, in some entities, ectopic lymphoid structures. These mechanisms are largely defined in experimental autoimmune uveitis and form the basis for extrapolating human pathogenesis. Tissue-resident memory T-cells persist into remission and may influence relapse risk. Autoinflammatory uveitis arises from dysregulated innate pathways independent of antigen specificity. Infectious uveitis reflects direct intraocular infection or reactivation. Post-infectious inflammation may be sustained by antigen persistence or molecular mimicry. Paraneoplastic uveitis (autoimmune retinopathy) arises when anti-tumour immunity cross-reacts with retinal antigens. Therapy should mirror the dominant immunopathology. In infectious uveitis, clinicians first reduce pathogen load with targeted antimicrobials and then add anti-inflammatory therapy under antimicrobial cover; maintenance antivirals curb reactivation when indicated. In autoimmune disease, where Th1/Th17–macrophage circuits dominate, steroid-sparing treatment targets TNF and IL-6 pathways. In autoinflammatory forms, excess inflammasome/IL-1 signalling supports IL-1 blockade. Advances in humanised modelling will be key to defining condition-specific mechanisms and supporting the evolution of tailored interventions.

## Introduction

Uveitis is a broad term representing a complex group of intraocular inflammatory disorders that collectively constitute a leading cause of preventable blindness worldwide (1). Uveitis is associated with up to 10% of vision impairment in the US and Europe (2). The annual incidence ranges from 17 to 52 per 100,000 people, with the condition predominantly affecting young and middle-aged adults aged 20 to 50 years (3). This demographic predilection amplifies the socioeconomic burden of uveitis-leading to significant loss of productivity, increased healthcare costs, and reduced quality of life.

The pathogenesis of uveitis reflects remarkable immunological diversity, encompassing infectious, autoimmune, autoinflammatory, and masquerade etiologies. Infectious aetiology as a cause varies according to country of origin, with 11 to 21% in high-income countries, and up to 50% in low- and middle-income countries. Autoimmune uveitis frequently occurs in association with systemic inflammatory conditions, with 37 to 49% of uveitis cases in the US and Europe associated with systemic disease such as HLA-B27-linked spondyloarthropathies, sarcoidosis, Behçet’s disease, and juvenile idiopathic arthritis. Autoinflammatory conditions, characterised by dysregulated innate immune responses, represent a distinct category exemplified by conditions such as Blau syndrome and other monogenic inflammatory disorders (4).

Successful uveitis management increasingly depends on understanding the underlying immunopathogenic mechanisms (1). The eye’s unique immunological environment, characterised by immune privilege and specialised regulatory mechanisms, requires targeted therapeutic approaches. This review will provide an overview of ocular immunology and the immunopathogenesis of uveitis. We will examine how distinct immune mechanisms contribute to the various classifications of uveitis and analyse the molecular basis underlying both infectious and non-infectious inflammation. As long as human ocular tissue remains restricted in availability and humanised *in vitro* systems remain relatively novel, mechanistic research in uveitis has depended on animal models, and the concepts discussed throughout this review should be interpreted with this limitation in mind. This review will not cover mucosal immunopathology as it pertains to corneal disease, maintaining specific focus on mechanisms underlying uveal inflammation.

## Discussion

### Uveitis

Uveitis is an umbrella term to describe numerous ocular inflammatory conditions causing inflammation of the uvea, which consists of the iris, the ciliary body, which creates aqueous humor and changes the shape of the lens, and the choroid, which is the vascular lining of the eye. Uveitis is classified anatomically into four categories based on the primary site of inflammation. Anterior uveitis represents the most common form, accounting for 41 to 60% of cases, followed by posterior uveitis at 17 to 23%, intermediate uveitis at 9 to 15%, and panuveitis at 7 to 32%. Specific systemic diseases demonstrate predilection for particular anatomical locations; axial spondyloarthritis is associated with anterior uveitis in approximately 91% of affected individuals, whereas multiple sclerosis manifests predominantly as intermediate uveitis in roughly 80% of cases (1, 2).

Infectious aetiologies account for 11 to 50% of uveitis cases, with higher rates observed in low- and middle-income. Viral causes include herpes simplex virus, varicella zoster virus, cytomegalovirus, and human immunodeficiency virus. Bacterial causes include tuberculosis, syphilis, and Lyme disease. Toxoplasmosis represents the predominant parasitic aetiology, whilst fungal causes such as candidiasis are encountered less frequently. Non-infectious aetiologies constitute 52 to 79% of cases. Conditions with established systemic associations include sarcoidosis (a multisystem granulomatous disorder), Behçet’s disease (a systemic vasculitis characterised by recurrent oral and genital ulceration), Vogt-Koyanagi-Harada syndrome (a multisystem autoimmune disorder targeting melanocytes), juvenile idiopathic arthritis, tubulointerstitial nephritis with uveitis syndrome, and multiple sclerosis. The HLA-B27-associated spondyloarthropathies encompass axial spondyloarthritis, reactive arthritis, psoriatic arthritis, and inflammatory bowel disease-associated arthritis. Uveitic conditions without known systemic associations include Fuchs’ heterochromic uveitis, Posner-Schlossman syndrome, birdshot chorioretinopathy, multifocal choroiditis with panuveitis, and sympathetic ophthalmia, among others. No identifiable cause is established in 27 to 51% of presentations despite comprehensive investigation. Ocular trauma contributes to 5 to 20% of cases. Masquerade syndromes, accounting for 1 to 5% of presentations, comprise neoplastic processes and non-neoplastic entities such as ocular ischaemia. Drug-induced uveitis occurs in approximately 0.5% of cases and has been associated with immune checkpoint inhibitors, bisphosphonates, rifabutin, and fluoroquinolones.

### Ocular immune privilege

Early evidence of ocular *immune privilege* dates to 1873, when Dutch ophthalmologist *Aartus van Dooremaal* observed the prolonged survival of murine skin grafts placed into the anterior chamber of canine eyes, which led to the suggestion that the eye possessed the ability to suppress the immune rejection of foreign tissue (5). This phenomenon gained clinical relevance with *Eduard Zirm’*s 1905 bilateral full-thickness corneal transplantation performed without immunosuppression; of the two grafts, one failed early while the fellow graft remained clear (6). In the 1940s, *Peter Medawar* further expanded on the concept of immune privilege: skin allografts placed in rabbit anterior chambers survived even in hosts pre-sensitised to reject skin elsewhere (7). He attributed this to the eye’s lack of conventional lymphatic drainage and the blood–ocular barrier, proposing a passive mechanism of immunological ignorance in which donor antigens failed to reach draining nodes and circulating effector cells were impeded from entering the graft. He further argued that acceptance persisted so long as the graft remained avascular, with rejection following neovascularisation-a principle later borne out in corneal models where neovascularisation precipitated rejection (8, 9).

This notion of simple sequestration was challenged in the 1970s (5, 10, 11). *Henry Kaplan* and *Wayne Streilein* demonstrated that antigens introduced into the anterior chamber did in fact undergo host immune recognition, but provoked an atypical inflammatory response: serum antibody develops on schedule while delayed-type hypersensitivity (DTH) is selectively curtailed-an observation that was defined as anterior chamber-associated immune deviation (ACAID) (12, 13). In ACAID, ocular introduction of antigen elicits tolerance rather than a typical inflammatory response, mediated by two key populations of regulatory T-cells that work in concert to suppress inflammation. The first population consists of CD4^+^ T-cells that act as “afferent suppressors”-these cells, now recognised as part of the regulatory T cell (Treg) family, initiate the suppressive response by recognising the antigen and signalling that tolerance, rather than inflammation, is needed. The second population comprises CD8^+^ T-cells serving as “efferent suppressors”; these cells execute the suppressive function by directly inhibiting inflammatory responses at the tissue level. While antibody production continues, the response shifts away from strongly complement-activating IgG subclasses (notably IgG1 and IgG3 in humans) that would normally activate complement-a cascade of proteins that can cause tissue damage-towards the less inflammatory IgG4. At the time, a splenic requirement for ACAID was recognised: induction fails if the spleen is removed during the first 10 days after intracameral antigen, but not thereafter; likewise, removing the antigen-bearing eye within this window prevents ACAID-evidence for an early *oculo–splenic axis* in tolerance induction. Today, eye-derived F4/80^+^ antigen-presenting cells (APCs) are known to exit the eye and collaborate in the spleen with marginal-zone B cells and CD1d-restricted natural killer (NK) T-cells to generate the antigen-specific CD4^+^ and CD8^+^ regulatory T-cell circuits that underpin ACAID.

**Figure 1:**
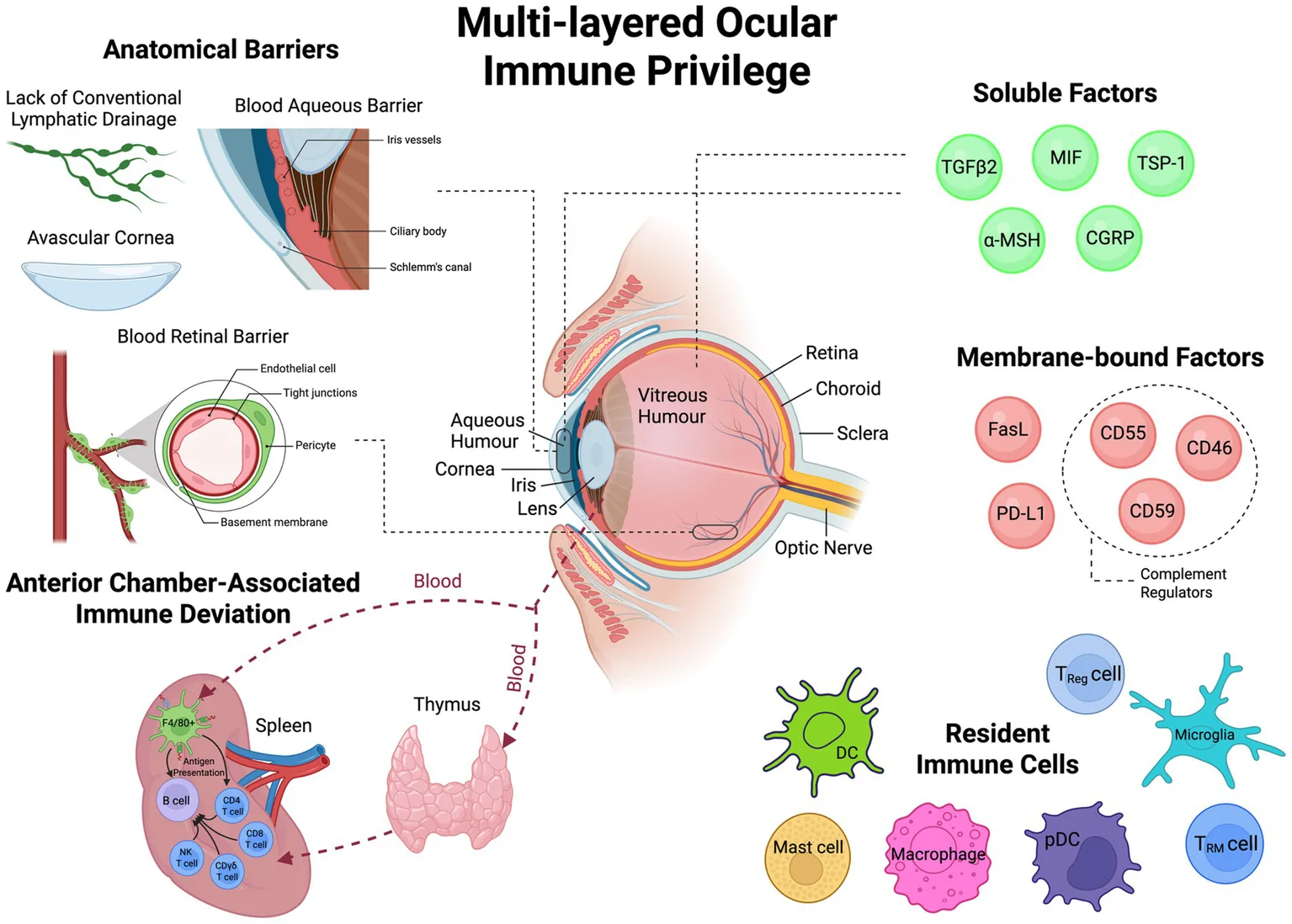
The multi-layered components that maintain ocular immune privilege. A schematic representation of the multi-layered physiological system which functions to maintain immune privilege within the eye. Anatomical barriers such as the blood-retinal barrier and blood-aqueous barriers, as well as the avascular nature of the cornea and lack of conventional lymphatic drainage physically separate the systemic immune system and the internal structures of the eye. The eye also possesses local immunosuppressive factors, including soluble factors and membrane-bound factors. Resident immune cells are found throughout the eye that constantly surveil the local microenvironment for potential pathogens. Systemic immune tolerance mechanisms such as anterior chamber-associated immune deviation (ACAID) provides an immunophysiological axis between the eye and the spleen which promotes antigen-specific tolerance within the intraocular environment. Created in BioRender.

From the 1990s onwards, ocular immune privilege came to be defined as layered: (i) anatomical compartmentalisation via the blood–aqueous and blood–retina barriers; (ii) a locally immunosuppressive milieu-notably aqueous humour TGF-β2 and *α*-MSH, and ocular expression of complement regulators (CD46/CD55/CD59); and (iii) systemic tolerance exemplified by ACAID with antigen-specific CD8^+^ regulatory T-cells (Table 1) (5, 6, 11, 14–60). These tiers aim to “throttle” intraocular inflammation and maintain immune quiescence (28). Crucial research throughout the 2010s deepened understanding around signalling pathways involving membrane-bound immunoregulatory molecules such as PD-L1 and FasL, and identified the role of Tregs and the wider adaptive immune system in maintaining intraocular immune quiescence (Figure 1) (17).

**Table 1:** Overview of the mechanisms maintaining ocular immune privilege.

Category	Mechanisms	Function
Anatomical and physical barriers	Bloods-aqueous barrier	Iris endothelial tight junctions and non-pigmented ciliary epithelium restrict antigen/protein flux from plasma to aqueous humour and limit leucocyte entry.
	Blood-retina barrier (BRB)	Excludes blood-borne antigens. *Outer BRB*: RPE tight junctions segregate the neural retina from the fenestrated choriocapillaris. *Inner BRB*: Non-fenestrated retinal endothelium, pericytes, astrocytes and Müller cells limit leucocyte and solute entry.
	Avascular cornea	Absence of blood and lymphatic vessels (“angiogenic privilege”) limits leucocyte ingress and afferent antigen drainage.
	Limited lymphatic drainage	Intraocular tissues lack conventional lymphatics. Sparse dural/meningeal lymphatics along the optic nerve link to cervical lymph nodes; curtailed antigen/APC egress reduces systemic activation.
Soluble Factors	TGF-β2	Promotes Treg induction/function, suppresses NK cytotoxicity, and dampens APC activation.
	α-MSH	Promotes Treg induction; signals via melanocortin receptors → ↑cAMP → PKA signalling, which suppresses NF-κB and pro-inflammatory cytokines. Supports BRB integrity and is anti-angiogenic in the cornea.
	MIF	Inhibits NK cytotoxicity and suppresses macrophage IL-12 production and Th1 polarisation.
	CGRP	Suppresses macrophage NO production and pro-inflammatory cytokines.
	TSP-1	Activates latent TGF-β2, suppresses effector T-cells, skews APCs toward a tolerogenic phenotype and dampens retinal microglial activation.
Membrane-bound Factors	FasL (CD95L)	Expressed on corneal epithelium/endothelium, iris–ciliary body and RPE; triggers apoptosis of Fas^+^ effector T-cells and neutrophils, limiting inflammation and neovascularisation.
	PD-L1	Expressed on corneal epithelium/endothelium, iris–ciliary body and RPE; engages PD-1 on T-cells to inhibit proliferation and cytokine production and to promote Treg.
	Reduced MHC	Corneal stromal APCs often display an immature phenotype with low MHC II and reduced co-stimulatory molecules (CD80/CD86, CD40), rendering them relatively quiescent.
	Complement Regulatory Proteins	Endothelium and RPE express CFH, CFI, CD46 (MCP), CD55 (DAF) and CD59 to accelerate decay of convertases, act as cofactors for C3b/C4b inactivation and block MAC formation, limiting bystander lysis and inflammation.
	Non-classical MHC Molecules	Expression of atypical class-Ib antigens (HLA-G, HLA-E in humans) provides inhibitory signals to NK cells.
Metabolic immune regulation	IDO1	Catalyses tryptophan degradation to kynurenines that activate the aryl hydrocarbon receptor (AhR), promoting effector T-cell apoptosis and Treg induction.
	Arginase	Depletes extracellular L-arginine, limiting T-cell proliferation; demonstrated in murine models to dampen local immune responses.
Immune cells and tolerance mechanisms	Regulatory T Cells	Suppress effectors via granzyme/perforin and anti-inflammatory cytokines; modulate DCs (including via IDO) and help prevent relapse in ocular tissues.
	F4/80 + macrophages	Migrate to the spleen with marginal-zone B cells and NKT-cells to initiate ACAID through CD4^+^/CD8^+^ Treg circuits.
	Regulatory dendritic cells	Induced by ocular factors (TGF-β2, α-MSH, VIP); adopt an IL-10^+^, low-co-stimulation phenotype and traffic to the spleen to generate CD8^+^ Tregs and support immune deviation.
	Resident microglia	Clear debris, coordinate with the BRB and contribute to immune exclusion.
	NK cells	Classical NK cells contribute to deletion of Th1 effectors. CD1d-restricted NKT-cells secrete IL-10 and support generation of CD8^+^ Tregs as part of the ACAID pathway.
Systemic tolerance	ACAID	Ocular F4/80^+^/CD11b^+^ APCs carry antigen to the spleen and, together with CD1d-restricted NKT-cells and marginal-zone B cells, induce CD4^+^ (*afferent*) and CD8^+^ (*efferent*) Tregs. IL-10, TGF-β and Qa-1/HLA-E–CD94/NKG2A interactions are implicated. Outcome: DTH suppression, antibody isotype skewing and antigen-specific systemic tolerance; early splenic involvement is required.

A summary of the anatomical, cellular, molecular, and systemic contributors to immune privilege in the eye. It highlights the coordinated function of physical barriers (e.g., the blood–aqueous and blood–retina barriers), immunosuppressive soluble and membrane-bound factors, metabolic regulation, and tolerogenic immune cells. Together, these mechanisms reduce antigen entry, inhibit inflammatory responses, and facilitate regulatory tolerance; ACAID, Anterior Chamber-Associated Immune Deviation; AhR, Aryl Hydrocarbon Receptor; APC, Antigen-Presenting Cell; BRB: Blood–Retina Barrier; CGRP, Calcitonin Gene-Related Peptide; C3/C5, Complement Components 3 and 5; CD, Cluster of Differentiation; CFH/CFI, Complement Factor H/I; DAF, Decay-Accelerating Factor (CD55); DC, Dendritic Cell; DTH, Delayed-Type Hypersensitivity; IDO1, Indoleamine 2,3-Dioxygenase 1; IL, Interleukin; LNs, Lymph Nodes; MAC, Membrane Attack Complex; MC-R, Melanocortin Receptor; MCP/DAF, Membrane Cofactor Protein/Decay-Accelerating Factor; MHC, Major Histocompatibility Complex; MIF, Macrophage Migration Inhibitory Factor; NKT, Natural Killer T Cell; NK, Natural Killer Cell; PD-1/PD-L1, Programmed Death Receptor-1 and its Ligand; RPE, Retinal Pigment Epithelium; TGF-β2, Transforming Growth Factor Beta-2; Teff, Effector T cell; Treg, Regulatory T cell; TSP-1, Thrombospondin-1; VIP, Vasoactive Intestinal Peptide (5, 9, 11, 14–60).

### Models of disease

**Figure 2:**
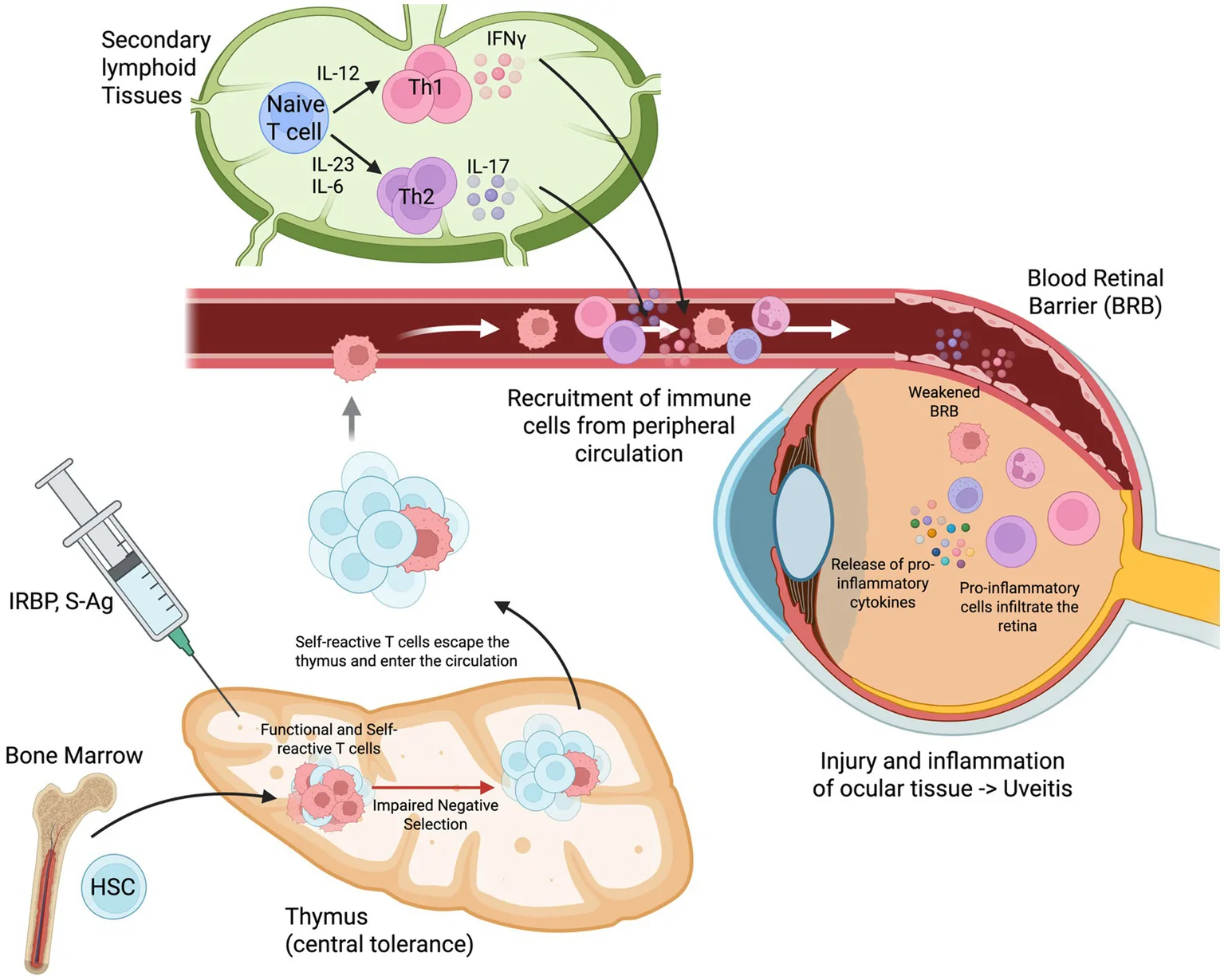
Mechanisms underlying experimentally induced autoimmune uveitis. Immunization with IRBP or S-Ag initiates the autoimmune cascade in the bone marrow and peripheral lymphoid tissues. During thymic development, impaired negative selection allows self-reactive T-cells to escape central tolerance mechanisms and enter the peripheral circulation. In secondary lymphoid tissues, naïve T-cells encounter retinal antigens and differentiate into effector T helper subsets: Th1 cells (producing IFN-γ in response to IL-12) and Th17 cells (producing IL-17 in response to IL-23 and IL-6). These activated autoreactive T-cells are recruited from the peripheral circulation and cross the blood-retinal barrier. The infiltration of pro-inflammatory immune cells into the retina causes BRB breakdown, local release of pro-inflammatory cytokines, and subsequent injury and inflammation of ocular tissues, culminating in uveitis. HSC, haematopoietic stem cell; IL, interleukin; IFN-γ, interferon-gamma; Th, T helper cell; IRBP, interphotoreceptor retinoid-binding protein; BRB: blood-retinal barrier. Created in BioRender. https://BioRender.com/p3kvfev.

Uveitis studies often use *in vivo* animal models due to the ethical considerations, limited supply, and risks of obtaining fresh biopsies of untreated uveitic human patients with active disease. Fixed ocular specimens may be used to confirm expression of certain markers, however are limited in their representation of the chronological progression of uveitis. Patient studies commonly use peripheral blood for established clinical trials; however, this is also limited in reflecting a single timepoint in uveitis (61). Although no animal model by itself represents the complexity of human uveitis, they have been instrumental in understanding immunopathogenesis throughout the disease time course (62). Various *in vivo* models of uveitis mimic the effects of different types, timeline durations, and severity of human uveitis; therefore due to its heterogeneity, specific models can be used that best fit the research question. For posterior non-infectious uveitis, one of the main models used in research is experimental autoimmune uveitis (EAU). EAU utilises various purified retinal autoantigens (S-Ag, interphotoreceptor retinoid-binding protein-IRBP, rhodopsin/opsin, phodusin, and recoverin) that can be induced using active immunisation or adoptive transfer, which results in a localised CD4 T cell driven disease (Figure 2) (61). Most EAU studies are conducted with rodent models due to their genetic susceptibility to a larger range of autoantigens that characterise the pathological features of the disease. Use of various disease induction methods such as active immunisation of autoantigens or adoptive transfer of activated pathogenic CD4 cells into naïve mice may also affect severity of disease (63).

Anterior uveitis has been most commonly studied using endotoxin-induced uveitis (EIU) which involves a systemic injection of bacterial endotoxin that triggers an acute innate-driven response in the anterior chamber. Although there is no human equivalent of EIU, this has been integral in understanding acute inflammatory responses in the eye (61, 64). Other models using melanin antigenic extracts are also used to induce experimental autoimmune anterior uveitis however are far less popular (64). Together, EAU and EIU are tools to comprehend the immunopathogenesis of uveitis and are commonly used as frameworks for emerging therapeutic developments.

### Innate immunity in the eye

**Figure 3:**
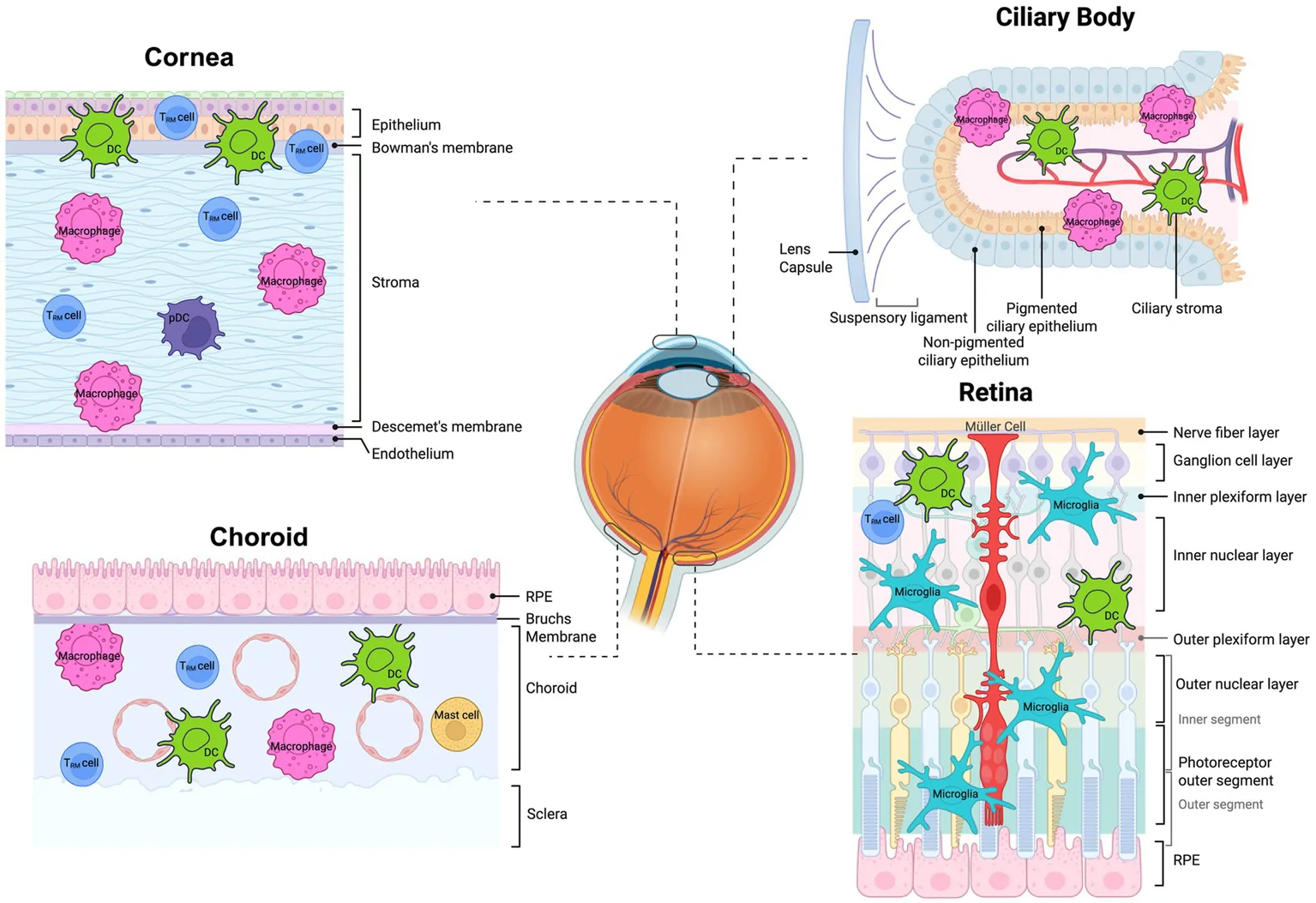
Tissue-resident immune cells present within various micro-environments within the eye. Figure showing resident immune populations present in healthy normal eyes. Trm cell - tissue-resident memory cell DCs - dendritic cells, pDCs, plasmacystoid dendritic cells. Created in BioRender. Han (2025) https://BioRender.com/c7qj6z2.

Despite ocular immune privilege, there are various immune cells that are resident to the eye. These cells are crucial in maintaining homeostasis as well as triggering an immune response (Figure 3).

#### Pattern recognition and early response

Innate immunity describes the immediate, non-specific inflammatory response triggered by non-self cells that bind to germline-encoded pattern recognition receptors (PRRs) present on various cell surfaces. These receptors may either bind to pathogen-associated molecular patterns (PAMPs), such as lipopolysaccharides, or to danger-associated molecular patterns (DAMPs), which are released as waste products from damaged cells, including heat shock proteins and fragments of the extracellular matrix (65). PAMPs are not produced by host cells, enabling the innate immune system to identify non-self entities, while DAMPs signal tissue damage regardless of its cause. Additionally, PAMPs are a crucial component of microbial survival and hence undergo few mutations, making them ideal targets for identification (66). Four main PRR families have been identified: transmembrane proteins, including Toll-like receptors (TLRs) and C-type lectin receptors (CLRs), and cytoplasmic proteins, including RIG-I-like receptors (RLRs) and NOD-like receptors (NLRs) (67). Among these, TLR signalling has been studied most extensively, and its inflammatory responses can be amplified by co-receptors such as CD14 or scavenger receptors such as CD36 (65). Other PRRs likewise rely on specific adaptor or accessory proteins, such as ASC for NLRs, to regulate downstream signalling.

TLRs are expressed in both immune and non-immune cells throughout ocular tissues, but the specific subtypes and their expression levels vary between sites (68). This variability supports immune privilege by enabling selective TLR-mediated inflammatory responses that protect against genuine pathogens while preventing unnecessary damage from harmless commensal flora at the ocular surface (69, 70). For example, in the human corneal epithelium, TLR4 and TLR5 are expressed in basal and wing cell layers but are absent or expressed at very low levels in the apical epithelial layers, which reduces the likelihood of reacting to surface-resident, non-pathogenic bacteria (71). Binding of TLRs to their respective ligands activates NF-κB signalling, leading to the release of pro-inflammatory cytokines such as TNF-*α*, IL-1β, and IL-12. These cytokines in turn activate downstream pathways involving antigen-presenting cells (APCs), including macrophages and dendritic cells, amplifying the immune response (70, 72). Although the eye possesses several mechanisms of immune privilege (Table 1), excessive or simultaneous activation of multiple TLRs can disrupt ocular homeostasis and contribute to pathologies such as uveitis (69).

#### Retinal microglia in immune surveillance and neuroinflammation

Originating from the primitive haematopoiesis of the yolk sac, microglia are specialised resident macrophage-like cells of the central nervous system (CNS) located within the inner retina (73), where they perform diverse functions, including maintaining neuro-retinal homeostasis through neurogenesis, phagocytic clearance of cellular debris, synaptic pruning, regulation of retinal angiogenesis, and modulation of inflammatory responses (73–75). As the principal resident immune cells of the retina, they conduct constant surveillance of their microenvironment by dynamically extending and retracting their processes to sample extracellular material and maintain neuronal contacts. Although they share core markers such as CD45, MHC class II, and CD68, microglia in different ocular regions can display distinct phenotypes: cells at the optic nerve head often exhibit a more macrophage-like profile suited to phagocytosis and debris clearance, whereas those in the ciliary body adopt a dendritic-like profile with greater emphasis on antigen presentation (76, 77).

Microglia can transition from a homeostatic state to a pro-inflammatory state in response to a variety of stimuli, including tissue injury, infection, neurodegenerative processes, and changes in the extracellular environment (78). In response to DAMPs or PAMPs, microglial PRRs-including TLR1, TLR2, TLR4, NLRs, CLRs, and CD14-are activated and promote phagocytosis (79–81). Microglia may also be activated by retinal neuron signals such as CX3CL1, CCL2, TGF-*β*, and nerve growth factors (82–85). Microglial activation is associated with upregulation of interferon regulatory factor-1 (IRF1), which drives pro-inflammatory gene expression (86, 87). Microglia initiate and amplify the inflammatory cascade by releasing key pleiotropic cytokines: TNF-*α* (regulated by TNFR1 and TNFR2) and IL-1β (regulated by IL-1R1 and IL-1R2) (88–90). These cytokines act in autocrine and paracrine loops to promote microglial activation and proliferation and can induce retinal neuronal injury via mitochondrial dysfunction and oxidative/nitrosative stress (91, 92). TNF-α and IL-1β initiate the neuroinflammatory cascade by upregulating endothelial adhesion molecules, such as ICAM-1 and VCAM-1, on retinal vessels (93). This endothelial activation compromises the Blood-retina barrier, promoting leukocyte adhesion and transendothelial migration into the retina (94). Recruitment is further amplified by chemokines produced within the retina-CCL2 and CXCL10-from microglia, Müller glia, retinal pigment epithelial cells. IL-1β also induces other pro-inflammatory factors such as cyclo-oxygenase-2 (COX-2) and inducible nitric oxide synthase (iNOS/NOS2), increasing ROS and NO and thereby exacerbating retinal cell toxicity.

#### Tissue-resident macrophage immunoregulation and activation

Macrophages are a key component of the innate immune system, present throughout the eye, and are highly adaptable to their environment, mounting protective or pathological responses (95, 96). Along with retinal microglia, ciliary body and corneal macrophages have been shown to originate from embryonic precursors in the yolk sac, but express differences in dependency levels on bone marrow-derived cells after birth, potentially explaining the differing compartment-specific turnover rates (97). Traditionally, macrophages have been classified into the M1-M2 polarisation paradigm (98). M1-polarised (classically activated) macrophages are monocyte-derived, activated by microbial stimuli such as lipopolysaccharide (LPS) and IFN-*γ* and secrete inflammatory mediators (e.g., TNF-*α*, IL-1β, IL-6) (99). By contrast, M2 macrophages are tissue-resident, anti-inflammatory cells stimulated by cytokines such as IL-4 and IL-13, secrete anti-inflammatory cytokines (e.g., IL-10 and TGF-*β*), and contribute to tissue repair and angiogenesis (VEGF, PDGF) (99). It is now accepted that the M1/M2 classification is oversimplified, with macrophages instead recognised as existing along a spectrum of activation states that are better defined by their gene-expression profiles and context-dependent functions (100).

Constituting approximately 10–15% of total cells in non-inflamed eyes, tissue-resident macrophages are important in immune surveillance and tissue remodelling (101). The specific roles of tissue-resident cells vary to suit the functional needs of their tissue microenvironment. Within the iris, ciliary body, and choroid, one of the main roles of macrophages is the phagocytosis of extracellular waste, foreign materials, and apoptotic cells, including melanin granules released from damaged pigment epithelium, thereby preventing their accumulation in the anterior chamber and maintaining a clear visual axis (102, 103). Macrophages present in the choroid express MHC class II and CD86 indicating their role in antigen presentation (103, 104). Detection of potential threats via PRRs or cytokine/chemokine/growth-factor receptors activates NF-κB and STAT signalling, reprogramming macrophages toward pro-inflammatory M1-like states (105). M1-type macrophages secrete large quantities of pro-inflammatory cytokines driving inflammation, endothelial activation, and recruitment of leukocytes (106, 107). Macrophages are also responsible for the xenophagy - a selective form of autophagy that targets intracellular pathogens and degrades their products when fused with lysosomes (105).

Hyalocytes are a mononuclear phagocyte subset residing at the vitreoretinal interface that plays a key role in vitreous metabolism and homeostasis by producing extracellular matrix components including Secreted Phosphoprotein 1 (SPP1) (108). These cells also maintain the immunosuppressive microenvironment to preserve vitreous transparency and visual function, shown by the expression of *α*-MSH, CD86, CD46, and TGF-β2, which induce regulatory T-cells (109). Contributing to immune surveillance, hyalocytes utilise their projections. When inflammation is triggered, hyalocytes abundantly express MHC, and facilitate the removal of extracellular debris (110).

#### Neutrophil recruitment and effector mechanisms

Neutrophils are short-lived polymorphonuclear phagocytes that are scarce in healthy ocular tissues beyond the surface due to the blood–aqueous and blood–retinal barriers, but are rapidly recruited as first responders during acute inflammation or infection. Within minutes of exposure to small microbes, neutrophils initially react through phagocytosis and opsonisation (50). When phagocytosis alone is insufficient against larger pathogens or biofilms, neutrophils degranulate and may undergo NETosis, releasing neutrophil extracellular traps (NETs)-web-like networks of decondensed chromatin decorated with histones and granular proteins-that physically ensnare microorganisms (111, 112). NETosis contributes to microbial death but is also implicated in autoimmune and autoinflammatory diseases (53). NETosis can be triggered by diverse stimuli, including NO, ROS, LPS, autoantibodies, cytokines (e.g., IL-1β, IL-6, IL-8, TNF-*α*), and interactions with activated platelets or vascular endothelial cells (113, 114). Degranulation and NETs together deliver antimicrobial peptides and proteases (e.g., defensins, elastase), MPO, and matrix metalloproteinases to the extracellular milieu, thereby degrading microbial components, immobilising pathogens, and amplifying recruitment of other immune cells (115). Although neutrophils are predominantly part of the innate immune response, they also modulate adaptive immunity (116, 117). By interacting with antigen-presenting cells, they influence T cell proliferation and enhance CD8^+^ T cell responsiveness to receptor stimulation (118). Neutrophils also aid in B cell activation to produce antigen-specific antibodies through IL-10 and IL-21 as well as the opsonisation of pathogens (119, 120).

#### Dendritic cell maturation and adaptive immune activation

Dendritic cells (DCs) are present in the cornea and choroid, but are sparse in the healthy retina and optic nerve head, where their numbers increase with injury or inflammation, and they play a key role in antigen presentation, immune surveillance, and activation of naïve T lymphocytes (121–123). They bridge innate and adaptive immunity by capturing antigen, migrating to draining submandibular and cervical lymph nodes, and priming naïve T lymphocytes. DCs are described along two intersecting dimensions: subset identity and maturation/functional state.

Subset-wise, conventional/classical DCs comprise cDC1 and cDC2, while plasmacytoid DCs (pDCs) form a distinct group. cDC1 (IRF8-dependent) specialise in cross-presentation to CD8^+^ T-cells and predominantly promote Th1/cytotoxic responses through superior IL-12 production. cDC2 (IRF4-dependent) are specialised for presenting exogenous antigens via MHC II to CD4^+^ T-cells and can polarise Th2 or Th17 responses, depending on environmental signals and cytokine milieu. pDCs are rare at baseline in healthy ocular tissues but are recruited during inflammation from the circulation/secondary lymphoid tissues (124). pDCs sense nucleic acids (TLR7/9) and are hallmark producers of type I interferons (especially IFN-α/β) (125, 126). They can also secrete TNF-α, IL-6, IL-10, IL-12 and chemokines such as CXCL10, CCL3, CCL4, and-upon activation-upregulate CD40, CD80, CD86, CD54, and MHC II to enhance (albeit less efficiently than cDCs) T-cell priming (126–129). Depending on cues, pDCs may amplify inflammation or promote tolerance, including Treg induction (130, 131). Understanding these subset-specific functions has therapeutic implications-cDC1 are targets for cancer immunotherapy due to their cross-presentation capacity, while modulating cDC2 responses may be relevant for controlling autoimmune inflammation.

Independently of subset, immature dendritic cells (imDCs) specialise in antigen capture through macropinocytosis, C-type lectin- or Fc receptor-mediated endocytosis, and the engulfment of apoptotic bodies (132–135). When imDCs detect microbial PAMPs, tissue-injury DAMPs, or licensing cues such as TNF-*α*/IL-1β or CD40L from activated T-cells, they differentiate into mature DCs (mDCs). Endocytosis decreases while processing and cross-presentation increase; MHC I/II, CD80/CD86, CD40, CD83, and CD54/HLA-DR are upregulated; and CCR7 is induced, directing migration via lymphatics to the eye-draining preauricular/submandibular nodes (136). DCs present peptides–MHC class I and II complexes on their surface for days and migrate to the submandibular eye-draining lymph node for recognition. The presence of both MHC classes enables activation of CD4^+^ and CD8^+^ T-cells, and DCs are exceptionally potent APCs-single cells can prime hundreds to thousands of T-cells (137, 138). Through IL-12/IL-15/IL-18 and contact-dependent signals, mDCs activate NK cells to produce IFN-*γ* and to release granzyme B and perforin; reciprocally, NK-derived TNF-*α*/IFN-γ further condition DCs (139, 140). DC–T-cell cross-talk (CD40–CD40L plus cytokines) amplifies T-cell responses and reinforces DC maturation, mediated by co-stimulatory molecules (CD80/CD86). DCs can also activate γδ T-cells in draining nodes, whose cytokines further shape DC function (137).

Regulatory/tolerogenic DCs (regDCs) are a functional state rather than a separate lineage. In the eye, a regDC population increases during EAU recovery, and suppresses T-cell proliferation by deploying nitric oxide (iNOS/NO), IL-10, IDO, and PD-L1/PD-L2 (Table 1), thereby limiting γδ T-cell expansion and IL-17^+^, IRBP-specific responses. The aqueous humour further biases DCs toward tolerance, while semimature, CCR7-competent regDCs can still reach draining nodes and promote Treg induction. Together, these features position regDCs as key brakes on Th17-biased ocular autoimmunity.

### Adaptive immunity in the eye

#### Antigen presentation and activation

Adaptive immune responses within the eye include the cell-mediated immunity orchestrated by T-cells and humoral immune response mediated by B cells and antibodies (65). Under healthy conditions, ocular tissues contain very low quantities of T and B cells (141). Following infection or injury, antigen captured by macrophages and DCs is transported to draining submandibular lymph nodes, where naïve T and B cells are primed (141). As covered under innate immunity, APCs initiate naïve T-cell activation in secondary lymphoid organs and help maintain activated T-cells at inflamed sites (65). B cells also function as APCs by expressing MHC class II and co-stimulatory molecules; their antibody-secreting role is discussed later. APCs are predominantly present in the corneal epithelium and stroma, conjunctival stroma, iris, and ciliary body, with fewer resident APCs in the posterior segment (142). In the special case of ACAID, ocular macrophages and DCs can exit the eye and induce systemic tolerance via the spleen, consistent with limited conventional lymphatics in the anterior chamber (143). CD8^+^ T-cell receptors recognise peptide–MHC I, whereas CD4^+^ T-cell receptors recognise peptide–MHC II on APCs to initiate cell-mediated immunity (141). Tolerogenic APCs, conditioned by the immunosuppressive ocular microenvironment, drive non-inflammatory adaptive responses and can induce ACAID, wherein ocular APCs promote antigen-specific Tregs that suppress both Th1- and Th17-mediated inflammatory responses while maintaining immune privilege (144).

#### T lymphocyte differentiation and effector response

T lymphocytes are defined by T cell receptors (TCRs) that recognise peptide antigens bound to MHC molecules on APCs (145). This initiates the adaptive immune response to defend the organ from pathogens by triggering clonal expansion and effector differentiation. T-cells originate from the bone marrow and mature in the thymus, where they undergo phases of positive selection for self-MHC restriction and negative selection to eliminate strongly self-reactive clones (146). This process discards T-cells that react to self antigens whilst preserving T-cells with functional TCRs.

CD4^+^ T (helper) cells coordinate adaptive immunity by differentiating into effector subsets that secrete lineage-defining cytokines. Th1 cells secrete IFN-*γ*, which amplifies macrophage microbicidal functions for immune cell recruitment and promotes CD8^+^ T cell responses (147, 148). Th2 cells produce IL-4, IL-5, and IL-13, promoting defence against helminths, eosinophil activation, and B-cell antibody production (147, 148). Th17 cells secrete IL-17, driving neutrophil recruitment and tissue inflammation (149, 150).

Foxp3^+^ regulatory T-cells (Tregs)-predominantly CD4^+^-are central mediators of ocular immune privilege. In the eye, antigen-specific Tregs are induced via ACAID and by signals from ocular resident/pigment epithelial cells and the aqueous humour (151). Tregs suppress the immune system by a range of mechanisms: (i) granzyme B and and perforin-mediated cytolysis; (ii) release of immunosuppressive cytokines (e.g., IL-10, TGF-*β*, IL-35); (iii) modulation of APCs, including inhibition of DC maturation and downregulation of co-stimulatory molecules; and (iv) metabolic competition through expression of CD25 and IL-2 (152, 153). This results in inhibition of macrophages, DCs, and effector T-cells, creating an immunosuppressive environment (153). These cells also demonstrate high degrees of plasticity, allowing them to functionally adapt according to cues from local immune cells (154, 155).

CD8^+^ T (cytotoxic) cells are recruited to sites of injury, which amplifies inflammation and vascular leakage (156). Once activated, CD8^+^ T-cells kill via two principal pathways: the Fas ligand–Fas (CD95) death-receptor axis, which triggers apoptosis, and the perforin–granzyme pathway, in which perforin forms membrane pores that permit granzyme entry and caspase activation, culminating in DNA fragmentation by endonucleases (157, 158). CD8^+^ cells also secrete IFN-*γ*, which restricts viral replication, upregulates MHC class I, and activates/recruits macrophages as effector cells and APCs (159).

CD4^+^ and CD8^+^ tissue-resident memory T-cells (Trm) exist throughout the eye within the cornea, conjunctiva, retina, and uvea (160–163). Recognised by cell surface markers CD69 and CD103, Trm cells monitor the presented antigens and initiate rapid responses to re-infection by promoting recruitment of central memory CD8^+^ T-cells and other lymphocytes by local release of cytokines (164). In murine models of uveitis, Trm cells infiltrate the retina following barrier breakdown during severe inflammation. These cells express markers of tissue residency and persist within the anterior uvea after resolution of active disease (165).

In the posterior segment of the eye, populations of resident γδ T-cells have been identified which contribute to local tissue homeostasis, immune tolerance, and wound healing (162, 163). The unique heterodimeric TCR on *γ*δ T-cells allows recognition of antigens without MHC involvement (166). Despite being observed in low quantities in normal healthy eyes, these resident cells exhibit the intrinsic capacity to become pathogenic effectors. In the anterior compartment, 95% of resident γδ T-cells express CD69, and CD44^high^ and display a CD62L effector phenotype (167). Response to IL-23 overexpression activates this resident population into pathogenic effector cells, by release of various cytokines including IL-17A and IFN-γ (identified through CCR6 and CD27 expression respectively) to orchestrate an inflammatory response (168, 169). Clinical features observed in the elicited response mimic posterior uveitis, as well as cellular inflammation within the vitreous and anterior compartments (167).

#### B cells in humoral immunity and ocular homeostasis

The primary function of B lymphocytes is their role in humoral immunity, providing long-term protection through antibody-secreting plasma cells and memory B cells; they also act as antigen-presenting cells and secrete cytokines (e.g., IL-6, IL-10) that shape immediate responses (170). B cells undergo central tolerance in the bone marrow-via clonal deletion, anergy, and receptor editing-before emigrating to the spleen as transitional (T1/T2) cells to complete maturation into naïve follicular or marginal-zone B cells bearing functional B-cell receptors (BCRs), thereby maximising non-self recognition while limiting autoimmunity (171–173).

Mature naïve B cells recirculate through secondary lymphoid organs until they encounter antigen. B cells receive the first activation signal by two antigen categories: thymus-dependent antigens, and thymus-independent antigens. For thymus-dependent protein antigens, B cells bind native antigen via the BCR, internalise it, and present processed peptides on MHC class II to cognate T follicular helper (Tfh) cells, whose TCRs recognise the same peptide–MHC II complex displayed by that B cell. Tfh help-via CD40–CD40L and cytokines such as IL-4 and IL-21-drives germinal-centre reactions, namely class-switch recombination (i.e., IgM → IgG/IgA/IgE, to alter effector function without changing specificity) and somatic hypermutation (i.e., introducing targeted mutations that refine the binding site and increase affinity), generating long-lived plasma cells and memory B cells (145, 174). Upon re-exposure to antigen, memory B cells mount accelerated, higher-affinity secondary responses and can differentiate into plasma cells with reduced reliance on T-cell help (99, 175). Plasma cells sustain antibody secretion mediating neutralisation, complement activation, and Fc-receptor-dependent opsonisation, whereas membrane-bound immunoglobulin functions as the BCR and secreted immunoglobulin executes effector functions (173). By contrast, thymus-independent antigens-such as LPS from Gram-negative bacteria-can activate B cells without T-cell help via BCR cross-linking and innate sensors (e.g., TLR4 for LPS), typically eliciting rapid, largely IgM-dominated responses with limited class switching and memory.

Beyond antibody production, B cells function as professional antigen-presenting cells (MHC II with CD80/CD86) and as cytokine producers that shape immunity. Canonical B-cell–derived cytokines include TNF-*α* and lymphotoxin-α, which regulate T cell–dependent antibody production, influence dendritic-cell organisation, and support antimicrobial responses (176, 177). In certain contexts, B cells can also produce IFN-*γ* or IL-12, though this is stimulus- and species-dependent. Polarisation toward type 1 versus type 2 immunity is largely imposed by helper T-cell cues: Th1-associated signals (e.g., IFN-γ, IL-12) bias B-cell responses toward opsonising, complement-fixing antibodies and pro-inflammatory programmes, whereas Th2-associated signals (e.g., IL-4, IL-5, IL-13) favour eosinophil-associated immunity and class-switch patterns characteristic of allergic/mucosal responses (e.g., IgE and-together with IL-5-IgA), with subclass outcomes varying by species and tissue (178–180). Regulatory B cells (Bregs) counterbalance these axes by releasing IL-10, TGF-*β*, and IL-35, thereby attenuating macrophage and dendritic-cell activation and limiting leukocyte recruitment (181). In the eye, local TGF-*β* and IL-10 in the aqueous humour further bias toward quiescence, restraining both type 1 and type 2 effector pathways and supporting ocular immune homeostasis (182, 183).

### Uveitis pathogenesis

As obtaining human biopsies from untreated uveitic patients with active disease is unethical and not feasible, human models of uveitis are often end stage, fixed specimens. This causes limitations in fully understanding the pathogenesis of the disease and may be affected by various anti-inflammatory treatments.

### Autoimmune uveitis

The development of uveitis is dependent on the disruption of immune privilege resulting in an imbalance between tolerance-promoting and effector mechanisms within the eye. As thymic education and negative selection are not always fully effective, some self-reactive T-cells may escape elimination and enter the periphery. Normally, these cells are controlled through peripheral tolerance mechanisms, including presentation of ocular antigens in deep cervical and submandibular draining nodes and active immunoregulation. However, tolerance can be weak as retinal antigens are sequestered behind the endothelial BRB (184). Certain types of uveitis have genetic associations that increase susceptibility. Mechanistically, susceptibility reflects how efficiently ocular self-antigens are processed and displayed and how strongly T-cells respond. HLA complexes (the polymorphic class I and II proteins of the human MHC) present peptide antigens to T-cells and thereby shape recognition of self versus foreign. Although HLA typing has diagnostic utility, there are mixed opinions of its usefulness due to the low positive predictive value (185). Table 2 includes a non-exhaustive list of some HLA associations with various types of uveitis. Variants in ERAP1/ERAP2 alter peptide trimming, while different HLA allotypes favour distinct peptide sets; together, these determine what is shown at the cell surface. If autoreactive T-cells carry higher-avidity TCRs for those peptide–HLA complexes, activation thresholds are lowered despite baseline tolerance. In short, differences in peptide processing (ERAP), peptide display (HLA), and TCR sensitivity combine to increase the likelihood that ocular antigens are recognised and targeted (186). The classification of certain entities, such as HLA-B27-associated acute anterior uveitis, remains debated; evidence supports contributions from both adaptive autoimmune mechanisms (such as molecular mimicry) and innate autoinflammatory pathways (including HLA-B27 misfolding and IL-23/IL-17 axis activation). Regardless of mechanistic classification, initial management remains corticosteroid-based across these entities.

**Table 2:** HLA associations with uveitic diseases.

HLA association	Condition	Ocular manifestation	Notes
HLA-A29 HLA-A29:02	Birdshot chorioretinopathy	Bilateral posterior uveitis with hypopigmented choroidal spots	Strongest known MHC–uveitis association. Reported relative risk ~50–224 × for A29-positive individuals (very high NPV for excluding BCR in A29-negative patients). Mechanistically, A29 shapes the presented peptidome; historical work suggested retinal S-antigen–reactive T-cell responses in many BCR patients, although the dominant autoantigen(s) remain debated.
HLA-B27	Seronegative HLA-B27 spondyloarthropathies (Axial spondyloarthritis, Reactive arthritis, Psoriatic arthritis, Inflammatory bowel disease-associated arthropathy)	Unilateral recurrent acute anterior uveitis	Common but weaker association than A29-BCR. AAU occurs in up to ~1/3 of SpA patients. Proposed mechanisms include molecular mimicry (gut microbial antigens), HLA-B27 misfolding/UPR driving IL-23/IL-17 (Th17) pathways, and B27 heavy-chain homodimers engaging KIR3DL2 on NK cells and Th17-skewed T-cells. Antigen presentation by B27 is class I (CD8^+^ T-cell)-restricted.
HLA-B51	Behçet’s disease (uveitis, recurrent oral/genital ulceration and skin lesions)	Panuveitis with hypopyon and occlusive retinal vasculitis	Major genetic risk for Behçet. Strongest association in populations along the “Silk Road” (e.g., Turkey, Iran, East Asia); weaker effect sizes in many European and African populations.
HLA-DRB1*04:05 HLA-DR4 HLA-DQ4 HLA-DQA1*03:01	Vogt-Koyanagi-Harada syndrome (uveitis, neurologic [meningism], auditory [tinnitus], and skin [poliosis, vitiligo, alopecia] signs)	Bilateral granulomatous panuveitis with exudative retinal detachments	DRB1*04:05 is the most consistently replicated risk allele.
HLA-DR2	Multiple sclerosis	Intermediate uveitis	DR2/DR15 is a shared genetic risk for MS and intermediate uveitis; helpful for pathobiology but limited diagnostic utility.
HLA-DRB1 HLA-DQB1	Sarcoidosis (multisystem granulomatous disease, involving lungs, lymph nodes and skin)	Anterior, intermediate, posterior or panuveitis	DRB1*04:01 can be protective for systemic disease yet increase risk of ocular sarcoidosis, whereas DRB1*03:01 is linked to acute/resolving phenotypes.
HLA-DRB1	Juvenile Idiopathic Arthritis (JIA)	Chronic anterior uveitis	HLA testing has low PPV and does not replace phenotype-based screening (e.g., age at onset, ANA, JIA subtype).
HLA-DRB1*01 HLA-DQA1*01	Tubulointerstitial nephritis and uveitis (TINU)	Bilateral acute anterior uveitis	Supportive association only.
HLA-B7	Presumed ocular histoplasmosis syndrome	Chorioretinal scars with risk of choroidal neovascularisation	Supportive association only

The table lists selected HLA alleles/subtypes, the primary associated condition, the typical ocular manifestation(s), and practical notes on interpretation. These associations are supportive rather than diagnostic: effect sizes and allele frequencies vary by ancestry, and positive predictive value is generally low outside the right clinical phenotype. AAU, acute anterior uveitis; ANA, antinuclear antibody; BCR, birdshot chorioretinopathy; CNV, choroidal neovascularisation; JIA, juvenile idiopathic arthritis; KIR, killer immunoglobulin-like receptor; MS, multiple sclerosis; NPV, negative predictive value; PPV, positive predictive value; SpA, spondyloarthritis; Th17, T helper 17; TINU, tubulointerstitial nephritis and uveitis; UPR, unfolded protein response; VKH, Vogt–Koyanagi–Harada.

In many forms of non-infectious uveitis, pathology is primarily CD4^+^ T-cell–mediated. PAMPs or DAMPs activate innate pathways and license APCs, promoting differentiation of pro-inflammatory T-cell subsets (Th1/Th17). In healthy eyes, naïve T-cells are restricted by the BRB preventing cell entry into the retina and subsequently preventing retinal antigens from being recognised by circulating lymphocytes. However, once T-cells are activated, they become more invasive in nature due to the expression of adhesion molecules and production of granzymes (187, 188). In uveitis, cytokine production of TNF-*α* and IL-1*β* results in transient gaps between BRB junctions and upregulation of adhesion molecules such as P-selectin and ICAM-1 (189, 190). The resulting compromise of BRB integrity facilitates intravascular adhesion, transmigration, and local interactions between infiltrating lymphocytes and intraocular APCs (191, 192). As outlined above, dendritic cells prime uveitogenic CD4^+^ T-cells in draining nodes, whereas within the retina local APCs-principally microglia-provide restimulation that sustains inflammation (193, 194). Effector Th1 and Th17 cells produce IFN-*γ* and IL-17, activating retinal microglia and recruited macrophages, which amplify damage via TNF-*α*, IL-6, IL-1β, IL-12, and reactive oxygen/nitrogen species (195–197). Upregulation of Nos2 gene expression mediates apoptosis due to the release of NO–resulting in lipid peroxidation of cell membranes and consequently further damaging ocular tissue architecture (198). Activated macrophages also produce chemoattractant molecules that promote further leukocyte infiltration such as CCL3 and CCL4.

Plasma cells may amplify uveitic inflammation through local antibody production and, when the BRB is disrupted, by increased entry of circulating antibodies (199, 200). The mechanistic role of anti-retinal antibodies in non-paraneoplastic uveitis remains heterogeneous-more consistent with disease amplification than universal causation-whereas they are pathogenic in paraneoplastic retinopathies. Ectopic lymphoid structures (ELS) have been reported in chronic ocular inflammation and can organise B- and T-cell zones, supporting class switching and plasma-cell survival, although their frequency and functional impact differ across uveitis entities (200–203).

Regulatory cell populations have been identified through the whole progression of EAU but only become functional in limiting disease at later stages. Experimental autoimmune encephalomyelitis studies focusing on isolated Tregs from the CNS suggest this could be due to the suppressive inflammatory environment at peak disease, resulting in dampened capacity, impaired proliferation of Tregs, and poor inhibition of CD4+ proliferation (204, 205). T-cell exposure to its cognate antigen in the presence of immunosuppressive environmental signals such as TGF-*β* in ocular fluids results in development of a more regulatory role to suppress other effector T-cells (206). The immune privileged status of the eye allows *in vivo* promotion of ACAID and post-recovery tolerance mechanisms that generate CD4+ and CD8+ Treg cells (207). Expression of melanocortin 5 receptor (MC5r) on APCs is crucial to activate Treg cells that express PD-L1 (208, 209). Stimulation of PD-1 blocks the PI3K pathway which inhibits TCR signalling and subsequently suppresses the expression of effector function by antigen-specific T-cells (209, 210). Aside from Tregs, regulatory B cells were also found to be positively recruited to the retina during EAU, accounting for >40% of total B cells detected by flow cytometry (200, 211). These cells have a key role in regulation of autoinflammatory responses by inducing T-cell apoptosis through a PD-L1- or FasL-dependent mechanism (212, 213). Bregs were found to be the primary producers of IL-10 and IL-35 which were confirmed to have a role in promoting generation of Tregs and inhibiting IL-17 and IFN-*γ* secretion, thereby suppressing inflammatory responses in uveitis (208, 211, 214, 215). Regulatory NK subsets also increase during the resolution phase, contributing to contraction of inflammation via IL-10 (216, 217). Macrophages can restrain effector T-cells within the retina through iNOS-derived nitric oxide, inducing apoptosis; however, they may also contribute to bystander tissue injury (198).

EAU models show that despite clinical signs of resolution and total reduction in immune infiltrate, the eye never fully recovers to its pre-inflamed baseline as low grade retinal infiltrates and microstructural alterations in neural and vascular architecture persist (218, 219). Although local clearance of immune cells occurs to terminate inflammation to enter a resolution phase, certain immune cell types remain within the eye in a state of adapted homeostasis. Residual cells have been reported around the posterior lens capsule and include CD11b^+^CD68^+^ macrophages with an Arg1^+^/CD206^+^ “M2-like” immunoregulatory phenotype, as well as CD4^+^ Tregs whose FoxP3 expression increases toward late time points (e.g., day 35) compared with peak disease (220). In autoimmune uveitis, antigen-experienced CD4^+^ and CD8^+^ T-cells accumulate in the eye, and a subset adopts a tissue-resident memory phenotype (CD69^+^ with context-dependent CD103^+^) within the uveal tract that persists into clinical remission (165).

#### Genetic triggers

The interaction between genetic predisposition and environmental triggers is central to the onset of uveitis. As outlined above, HLA polymorphisms shape which ocular peptides are displayed to T-cells and modify risk; prominent examples include HLA-B27 with acute anterior uveitis, HLA-A29 with birdshot chorioretinopathy, and HLA-B*51 with Behçet’s disease (Table 2) (221). In parallel, ERAP influences MHC class I peptide trimming and interacts with specific HLA backgrounds, contributing to risk stratification in HLA-associated uveitis (222).

In addition to HLA predisposition, genome-wide association studies have shown there are also non-HLA genetic loci that modulate risk in autoimmune uveitis (221). For example, IL23R is a key genetic component of Th17 signalling and is implicated in the pathophysiology of Behçet’s and Vogt-Koyanagi-Harada disease (223). Additional immune-regulatory loci-STAT4, CCR1, IRF8, TNFAIP3 (A20)-have been linked in cohort- and disease-specific analyses. A20 haploinsufficiency, which normally acts as a brake on the NF-κB inflammation pathway, disinhibits NF-κB signalling, lowering activation thresholds for ocular autoimmunity (221, 224). Polymorphisms in cytokine/receptor genes (IL10, IL1A/IL1B, IL18R1–IL1R1, IL6R) are also implicated in the disease (221).

A useful tool for quantifying risk of developing uveitis is a genome-wide polygenic risk score (PRS). PRS has been shown to have a higher discriminatory capacity than HLA-B27 testing alone for predicting the manifestation of both seronegative spondyloarthropathies and uveitis development (221, 225).

#### Environmental triggers

Air pollution is a well-recognised environmental trigger for uveitis. Long-term exposure to high levels of particulate matter increases circulating IL-1β, IL-6, and GM-CSF (226, 227). A large-scale retrospective analysis has shown increasing concentrations of CH₄, THC, NOx, NO, and CO are associated with an increased risk of developing uveitis. However, the precise immunological mechanisms for these relationships are still incompletely understood (228). NOx pollution may induce lipid peroxidation and increase cellular oxidative stress (229). Noxious gases may also contribute to autoimmunity by promoting T-lymphocyte production/expansion, and epigenetic modification has been proposed as a driver of this inflammatory process (228, 230, 231).

Cigarette smoking is linked to the development of all anatomical subtypes of uveitis consistent with its established associations with other inflammatory disorders (232, 233). It is thought to act via a complex inflammatory process driven by oxidative stress from pro-inflammatory constituents of cigarette smoke. While nicotine may exhibit context-dependent anti-inflammatory effects, whole cigarette smoke is net pro-inflammatory, in part by promoting vascular inflammation through the generation of reactive oxygen species (234, 235). Exposure to cigarette-smoke extract increases vascular hydrogen peroxide production, activates NF-κB, GATA, PAX5, and Smad3/4, and up-regulates key pro-inflammatory cytokines, including IL-1β, IL-6, and TNF-*α* (233, 235, 236).

Other lifestyle factors have a direct link to the development of uveitis. Two key mechanisms have been identified through which the gut microbiome contributes to the development of autoimmune uveitis: first, by serving as an antigenic trigger through activation of retinal antigen-specific T-cells within the gut; second, by modulating the equilibrium between pro-inflammatory effector cells (Th1 and Th17) and regulatory T-cells (Tregs). Additionally, dietary antigens can damage retinal cells via cross-reactive mechanisms and mimicry, while physical exercise has demonstrated therapeutic potential in ameliorating uveitis symptoms through reduction of reactive oxygen species. Reducing white adipose tissue through regular exercise decreases the production of pro-inflammatory cytokines such as IL-6 and TNF-*α* (236).

### Autoinflammatory uveitis

Pathogenic variants affecting the innate immune system can lead to spontaneous inflammation or exaggerated responses to minimal triggers. These conditions frequently involve dysregulation of inflammasome components, leading to innate immune activation and inflammatory responses that are entirely independent of adaptive immune memory (4, 237). Inflammasomes are cytosolic multiprotein pattern recognition receptors that detect molecular danger signals like PAMPs and DAMPs (238). NLRP3 is among the best-characterised inflammasomes and activates in two phases. In the priming phase, NF-κB signalling upregulates NLRP3 and pro-IL-1β/pro-IL-18 transcription; in the subsequent activation phase, stimuli such as crystalline particulates, nigericin, or extracellular ATP drive inflammasome assembly, caspase-1 activation, and cleavage of these precursors to their mature cytokines (4). The resulting cytokines, particularly IL-1β and IL-18, amplify inflammation by recruiting neutrophils, activating the vascular endothelium, and increasing vascular permeability (239–241). Additionally, inflammasome activation triggers pyroptosis-a form of programmed cell death characterised by gasdermin D-mediated pore formation in the cell membrane, leading to cell lysis, release of pro-inflammatory intracellular contents, and further amplification of tissue inflammation (4, 241).

Gain-of-function variants in the NLRP3 gene, which encodes the cryopyrin protein, are associated with a group of disorders collectively referred to as cryopyrin-associated periodic syndromes (CAPS) (4, 242). Such syndromes disrupt the normal autoinhibitory mechanism in this pathway, causing uncontrolled inflammasome activation in the absence of PAMPs and DAMPs (4, 243). Excess IL-1β is central to pathogenesis, and ocular involvement-often chronic, granulomatous uveitis-is well described in familial cold autoinflammatory syndrome, Muckle-Wells syndrome, and neonatal-onset multisystem inflammatory disorder. Three IL-1 blockers are licensed for CAPS, the NLRP3 diseases: canakinumab, rilonacept, and anakinra.

Blau–Jabs syndrome illustrates how dysregulated innate immunity can drive autoinflammatory uveitis. It is a rare monogenic, autosomal dominant disorder caused by gain-of-function pathogenic variants in the NOD2 gene. Patients typically present with granulomatous uveitis, arthritis, and dermatitis in early childhood, reflecting constitutive activation of innate immune pathways. NOD2 is an intracellular pattern recognition receptor that detects muramyl dipeptide from bacterial peptidoglycan, triggering the activation of the NF-κB signalling pathway. Blau-Jabs syndrome causes elevated NOD2 protein expression, leading to overactivation of this downstream pathway thus the unwarranted increased production of pro-inflammatory cytokines (4). Although the precise mechanisms of autoinflammation remain incompletely defined, experimental data implicate interferon-*γ* as a key driver of ocular inflammation (244).

Autoinflammatory uveitis can also arise in polygenic conditions in which inflammasome pathways are overactive. Behçet’s is considered a disease with overlapping autoimmune and autoinflammatory uveitic processes: alongside adaptive Th1/Th17 responses, innate circuits involving the IL-1 family (e.g., IL-1β, IL-18) and NLRP3 inflammasome activation contribute to pathogenesis and ocular flares (245). Clinical responses to IL-1 blockade in refractory Behçet’s further support this axis, although disease is clearly multifactorial rather than IL-1–driven alone. Another example is systemic juvenile idiopathic arthritis. Trials of IL-1 blocking drugs have improved clinical symptoms in this disease, further evidencing the role of genes controlling IL-1 production and downstream inflammasome activation in polygenic autoinflammatory uveitis (246, 247).

### Infectious uveitis

**Figure 4:**
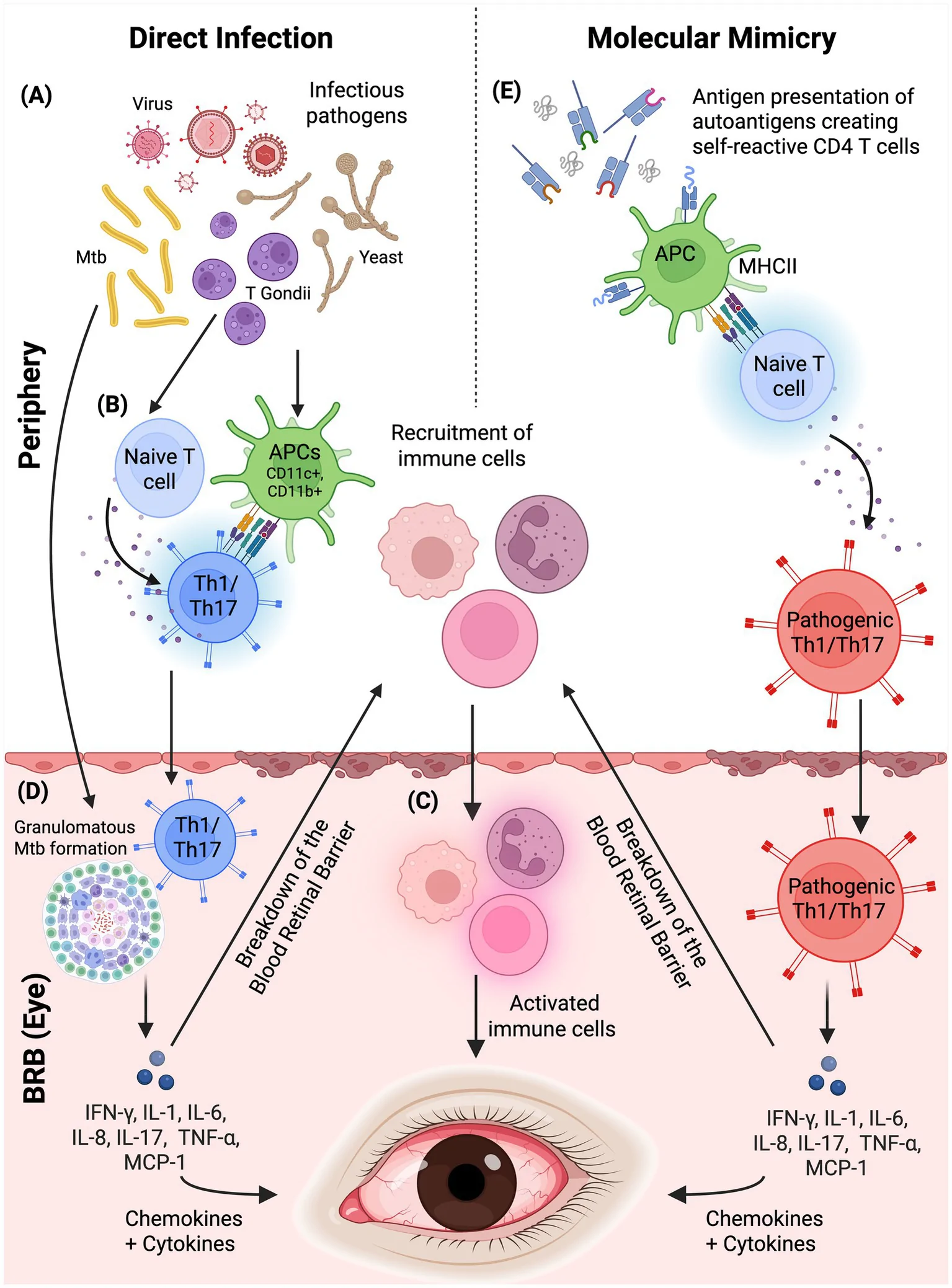
Mechanisms underpinning pathogen-triggered uveitis. Infectious uveitis can arise through two principal pathways. Left panel (Direct infection): **(A)** Various infectious pathogens, including viruses, bacteria (such as *Mycobacterium tuberculosis*), protozoa (*Toxoplasma gondii*), and fungi (yeast), can initiate ocular inflammation. **(B)** Pathogens are recognized by antigen-presenting cells (APCs; CD11c^+^, CD11b^+^), which prime naïve T-cells to differentiate into effector Th1/Th17 cells in the periphery. These activated T-cells, along with recruited innate immune cells (macrophages, neutrophils), breach the blood-retinal barrier (BRB). **(C)** Within the eye, activated immune cells release pro-inflammatory cytokines and chemokines (IFN-γ, IL-1, IL-6, IL-8, IL-17, TNF-α, MCP-1), amplifying local inflammation. **(D)** In tuberculosis-associated uveitis, Th1/Th17 responses drive characteristic granulomatous inflammation. Right panel (Molecular mimicry): **(E)** Structural similarity between microbial peptides and retinal self-antigens enables cross-reactive immune responses. APCs present microbial antigens via MHC class II to naïve T-cells; due to shared epitopes with ocular autoantigens, this generates pathogenic self-reactive Th1/Th17 cells. These autoreactive T-cells cross the BRB and release pro-inflammatory mediators, causing tissue damage in the absence of active intraocular infection. Both pathways converge on a common effector phase characterised by BRB breakdown, immune cell infiltration, and cytokine-mediated ocular injury. Created in BioRender. Han (2025) https://BioRender.com/n5uzs67.

Molecular mimicry is one mechanism by which infections can precipitate autoimmune (post-infectious or para-infectious) uveitis: structural similarity between microbial peptides and retinal self-peptides enables cross-reactive T-cell or antibody responses (Figure 4) (248–250). T-cell cross-reactivity is facilitated by the degeneracy in the recognition of peptides presented by MHC molecules. Peptide fragments with minimal sequence similarity can trigger T-cell activation if they preserve contact residues for MHC binding and TCR engagement epitopes (251–253). In this model, infection primes T-cells in peripheral tissues leading to activation of autoreactive T-cells. These cells migrate to the eye and cause intraocular inflammation by cross-reacting with autoantigens (254). Indeed, most cases of intraocular inflammation occur in the absence of preceding ocular trauma or infection. A range of bacterial, viral, fungal, and parasitic epitopes, as well as non-pathogenic antigens have been shown to induce such mimicry and are implicated in the pathogenesis of uveitis (254–258).

*Mycobacterium tuberculosis* (Mtb) and the *Bacille Calmette–Guérin* (BCG) vaccine can precipitate post-infectious/para-infectious uveitis via molecular mimicry, in addition to causing direct infectious uveitis. A notable case supporting this involved a patient receiving BCG immunotherapy for bladder carcinoma. Peripheral blood lymphocytes from this patient exhibited strong proliferative responses and cytokine release (IFN-*γ*, IL-6, IL-8, TNF-*α*, MCP-1) when stimulated with Mtb protein derivatives, as well as ocular antigens and retinal peptides. *In silico* analyses identified sequence homology between Mtb proteins and several retinal self-antigens, such as IRBP, and S-antigen, supporting a mimicry mechanism (254, 256). Beyond iatrogenic BCG exposure, TB-associated uveitis in the context of latent/systemic Mtb infection may arise through similar mechanisms (259–261). Two non-mutually exclusive models are proposed, involving persistence of mycobacterial products (PAMPs and nonviable bacillary components) that chronically stimulate innate immunity despite microbiological cure, and loss of self-tolerance with cross-reactive T-cell and antibody responses against retinal antigens (261–264). In tuberculosis-related uveitis, early inflammation can be precipitated by innate triggers from mycobacterial products, while chronic disease is sustained by adaptive T-cell responses (predominantly Th1/Th17 with IFN-*γ*, TNF, and IL-17). Granulomatous inflammation is characteristic-choroidal granulomas and granulomatous anterior uveitis are common. Aqueous studies in presumed TB uveitis report raised IL-6, CXCL9, CXCL10, and VEGF, consistent with ongoing T-cell recruitment and activation (261).

*Toxoplasma gondii* is an obligate intracellular protozoan that infects virtually all nucleated cells (265). Ocular disease is classically due to direct retinal infection-often reactivation of tissue cysts (bradyzoites) with local conversion to tachyzoites-producing necrotising retinitis and overlying vitritis. Following oral infection, the parasite likely disseminates throughout the host via a “Trojan horse” mechanism, whereby *T. gondii* infects CD11c^+^ dendritic cells and CD11b^+^ monocytes, hijacking their migratory capacity and may also transmigrate as tachyzoites across the BRB (266). Once in the retina/RPE, parasite replication triggers a type-1–skewed response with IFN-*γ* and IL-1β, barrier disruption, and leukocyte recruitment; infected retinal cells can undergo pyroptosis with inflammasome (e.g., NLRP1) activation, amplifying tissue injury (266–269). Although antigenic mimicry has been proposed-for example, sequence homology between parasite HSP70 and human HSP70-the dominant, well-supported mechanism in ocular toxoplasmosis remains direct infection (270).

*Treponema pallidum*, the infectious cause of Syphilis, can cause uveitis with a variety of clinical presentations ranging from anterior to posterior segment manifestations. The organism is capable of persisting in the immune-privileged environment of the eye during latent infection, aided by the organism’s low density of surface-exposed antigens and antigenic variation (271). Pathogenesis is primarily direct infection, with possible contributions from immune-complex vasculitis and molecular mimicry (271). In one epitope-mapping study, a highly immunoreactive sequence within the 47-kDa lipoprotein (TpN47)-PGTEYT411-416-shared motif homology with repeats in mammalian fibronectin, an extracellular-matrix glycoprotein expressed in trabecular meshwork, retina, and cornea (272).

Viral uveitis reflects direct cytopathic infection plus host-driven inflammation, with reactivation central for herpesviruses. After primary infection, HSV-1/2 and VZV enter latency in sensory ganglia and can reactivate to produce anterior uveitis, keratouveitis, or acute retinal necrosis. CMV can persist latently within ocular tissues such as the corneal endothelium and reactivate under local or systemic immunosuppression to cause anterior uveitis or retinitis. In Fuchs’ uveitis, evidence supports persistent intraocular rubella virus as a chronic, low-grade trigger. Even after virological control, inflammation may persist due to antigen persistence or immune reconstitution (273, 274). Reactivation is promoted by waning immunity and iatrogenic factors (e.g., corticosteroids, checkpoint inhibition), explaining relapsing courses in predisposed hosts (248, 252). The treatment philosophy mirrors immunopathogenesis. Because herpes viruses and CMV cause disease by reactivation and ongoing replication that trigger a disproportionate host inflammatory response, management first suppresses viral replication to reduce antigen load, then modulates the immune response to limit bystander tissue injury (1). Practically, this means initiating antiviral therapy upfront, and adding anti-inflammatory treatment (typically corticosteroids and cycloplegia for anterior disease) only after antiviral cover is in place. During convalescence, maintenance/suppressive antivirals are used in high-relapse phenotypes to prevent further reactivation, with duration tailored to the virus and host immune status.

#### Paraneoplastic and autoimmune retinopathies

Paraneoplastic retinopathies arise when anti-tumour immunity cross-reacts with retinal proteins. Entities include melanoma-associated retinopathy, bilateral diffuse uveal melanocytic proliferation, paraneoplastic vitelliform maculopathy, and cancer-associated retinopathy (CAR) (275). The syndromes are linked to a wide-range of underlying cancers. For CAR the most frequent primaries are small-cell lung cancer, gynaecological malignancies, and breast cancer (276–278). The underlying mechanism of paraneoplastic retinopathies overlaps with systemic autoimmunity via cross-reactivity and molecular mimicry between tumour antigens and normal host retinal self-antigens break tolerance, leading to pathogenic autoantibodies and, in some cases, T-cell responses (275, 279, 280). Well-characterised anti-retinal autoantibodies include anti-recoverin (targets a photoreceptor calcium-binding protein), anti-*α*-enolase (a glycolytic enzyme expressed in multiple retinal cells), and anti-TRPM1 (an ON-bipolar-cell ion channel) (280).

## Conclusion

The ocular immune system is specialised to protect against potential pathogens whilst limiting collateral damage. This is achieved through a combination of anatomical barriers such as the blood-retinal barrier, immunosuppressive molecules, and resident immune cells that regularly surveil its microenvironment. Uveitis describes the inflammation of the uvea and is one of the leading causes of visual impairment worldwide. Autoimmune uveitis reflects failure of tolerance and priming of Th1/Th17 responses that overcome barrier and regulatory checks. Autoinflammatory uveitis arises from dysregulated innate pathways that ignite tissue inflammation with minimal triggers. Infectious uveitis is driven primarily by direct intraocular infection or reactivation, with post-infectious immune sequelae (including molecular mimicry) contributing in selected settings. Key uncertainties include the dominant autoantigens in many entities, why some episodes resolve while others become chronic, how tissue-resident memory T-cells affect disease, the relative contribution of microbiome-driven signals, and which combinations of cytokines and cell states best define actionable endotypes. We also lack robust biomarkers to predict flare risk, therapeutic response, and long-term visual outcomes across ancestries and disease subtypes. Deeper mechanistic insight will enable precision, mechanism-guided care, restoring tolerance, and using local delivery to maximise ocular benefit with fewer systemic effects. Coupled with endotyping via genetics, imaging, and fluid biomarkers, this shifts management from blanket immunosuppression to tailored regimens that better preserve vision and reduce treatment burden.

## Glossary

Central Tolerance: The deletion or inactivation of autoreactive T and B cells during development in the thymus and bone marrow. This ensures that most lymphocytes capable of recognising self-antigens are eliminated before entering circulation.
Peripheral Tolerance: Mechanisms that suppress autoreactive lymphocytes that escape central deletion, including T regulatory cells, energy, and immune deviation. Critical for maintaining immune quiescence in immune-privileged tissues like the eye.
Immune Privilege: A specialised state in which the eye limits immune responses to prevent damage to vision-critical tissues. Maintained by physical barriers, local immunosuppressive factors, and systemic tolerance mechanisms.
Immune regulation: The dynamic modulation of immune responses to ensure a balance between defense and preservation of tissue integrity. In the eye, this includes secretion of anti-inflammatory cytokines and activation of regulatory T cells.
Immune surveillance: The continuous monitoring of ocular tissues by resident immune cells (e.g., microglia, macrophages) to detect infection, injury, or cellular stress.
Tissue-resident immune cells: Immune cells such as microglia, perivascular macrophages, and dendritic cells that are permanently located in ocular tissues and contribute to surveillance, repair, and inflammatory regulation.
Anterior chamber–associated immune deviation (ACAID): A form of systemic immune tolerance initiated when antigens enter the anterior chamber of the eye. Antigen-presenting cells drain to the spleen and induce regulatory T cell responses rather than pro-inflammatory ones.
Immune deviation: The phenomenon by which immune responses are skewed away from destructive pathways (e.g., Th1 or Th17) toward more tolerogenic ones (e.g., Th2, Tregs).
Innate immunity: The eye’s first line of immune defense. It involves non-specific mechanisms including microglia, macrophages, complement proteins, toll-like receptors (TLRs), and cytokine release. Innate responses are rapid but lack antigen specificity.
Adaptive immunity: A delayed but highly specific immune response involving T and B lymphocytes. In uveitis, autoreactive CD4 + T cells (especially Th1 and Th17 subsets) and antibody-producing B cells play a central role in disease pathogenesis.
Loss of immune privilege: Disruption of the ocular immune suppressive environment due to infection, trauma, or genetic predisposition, allowing immune cell infiltration and activation that can lead to uveitis.
Inflammatory priming: A state in which prior immune exposure, genetic risk, or systemic inflammation sensitises ocular tissue, lowering the threshold for future immune activation and inflammation.
