# Correction: The immunopathogenesis of uveitis

**DOI:** 10.3389/fmed.2026.1976368

**Published:** 2026-09-11

**Authors:** Jimin Han, James Harper, David A. Copland, Panayiotis Maghsoudlou

**Affiliations:** 1 Academic Unit of Ophthalmology, Translational Health Sciences, Bristol, United Kingdom; 2 National Institute of Health Research (NIHR) Moorfields Biomedical Research Centre, Moorfields Eye Hospital NHS Foundation Trust and UCL Institute of Ophthalmology, London, United Kingdom

**Keywords:** autoimmunity, HLA-ERAP axis, immune privilege, inflammasome, molecular mimicry, uveitis

The funder UK Medical Research Council, Developmental Gap Pathway Funding MR/Z50385X/1 to Copland DA was erroneously omitted. The correct funding statement reads:

The author(s) declared that financial support was received for this work and/or its publication. DAC was supported by the UK Medical Research Council, Developmental Gap Pathway Funding MR/Z50385X/1 and National Institute of Health Research (NIHR) Moorfields Biomedical Research Centre, Moorfields Eye Hospital NHS Foundation Trust and UCL Institute of Ophthalmology, London, EC1V 2PD, UK [Grant#: BRC4-03-RB414-405]. PM is an NIHR-funded Academic Clinical Lecturer.

The original version of this article has been updated.

